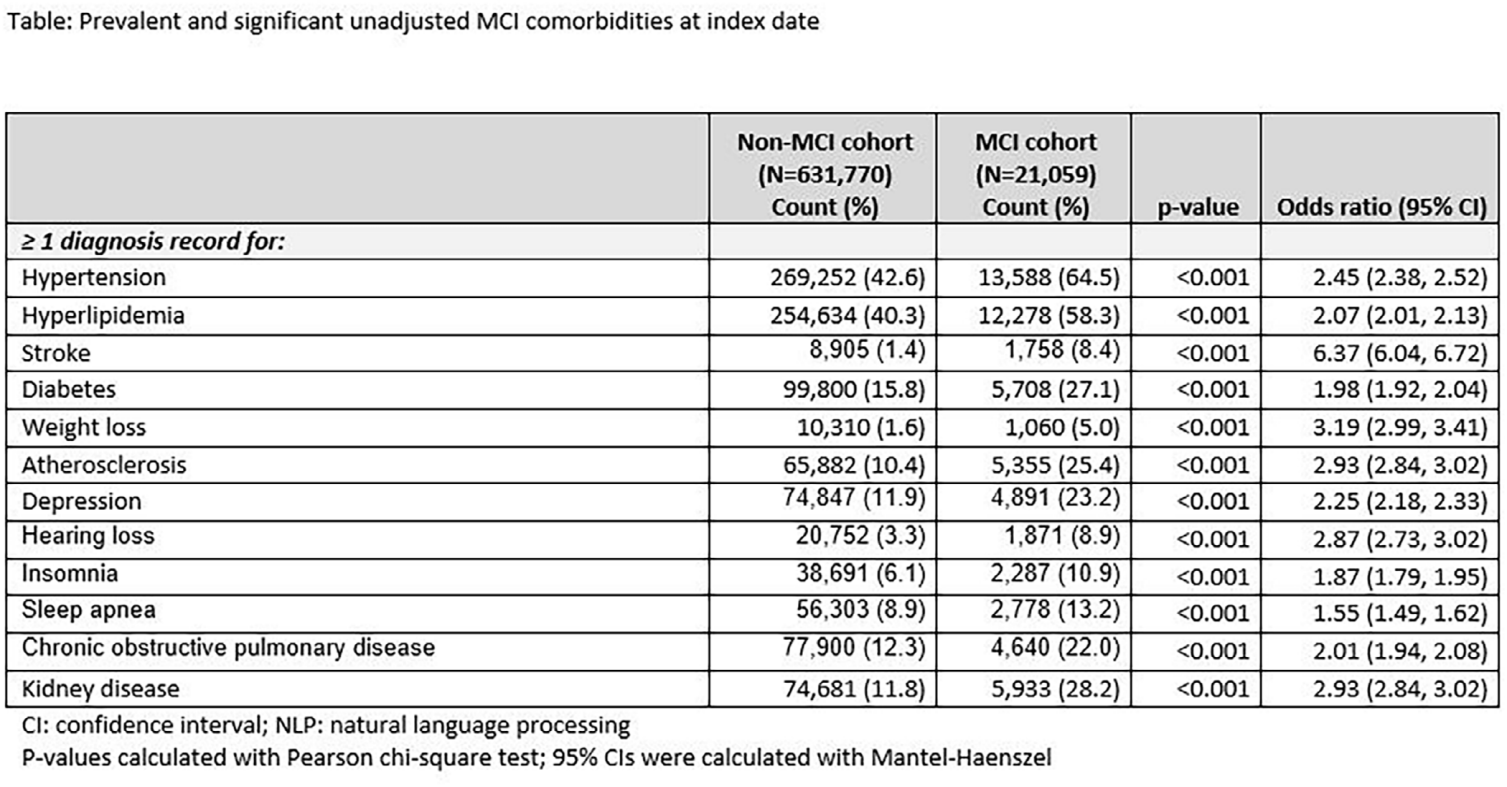# Risk Factors for Mild Cognitive Impairment: Prediction Models Developed with Electronic Health Record Data

**DOI:** 10.1002/alz.085564

**Published:** 2025-01-09

**Authors:** Gang Li, Viswanath Devanarayan, Rachel Halpern, Joanne Bell, Richard Batrla, Susan DeSanti, Feride H Frech, Jo Vandercappellen, Ara S Khachaturian, Richard Crislip, Jeffrey Meyerhoff, Saul Perea, Soeren Mattke, Harald Hampel

**Affiliations:** ^1^ Eisai Inc., Nutley, NJ USA; ^2^ Optum, Eden Prairie, MN USA; ^3^ Prevent Alzheimer’s Disease 2020, Inc., Rockville, MD USA; ^4^ OptumCare, Phoenix, AZ USA; ^5^ Optum Behavioral Health, Portland, OR USA; ^6^ University of Southern California, Los Angeles, CA USA

## Abstract

**Background:**

Timely identification of mild cognitive impairment (MCI) is key to early intervention. While primary care providers are the most likely entry point to detect early signs of MCI, their detection rates are low. Building upon a published study, we used electronic health records (EHR) to develop a clinically enhanced MCI risk prediction algorithm.

**Method:**

This retrospective analysis utilized the Optum EHR database, which includes records for more than 110M individuals across the United States. MCI and non‐MCI cohorts were identified from January 2016 – March 2021. Index date was first MCI diagnosis for MCI cohort and randomly selected diagnosis record for non‐MCI cohort. All individuals were observed 2 years before index date. Derived from EHR, potential predictors of MCI included health status (e.g., body mass index [BMI]), pre‐existing conditions identified with EHR diagnosis records and abstracted note records derived via natural language processing. Predictors of MCI will be identified using logistic regression, and an MCI risk prediction algorithm will be established using machine‐learning approaches based on those predictors.

**Result:**

Our sample included 21,059 and 631,770 individuals with and without MCI and mean age 71.1 and 59.1 years, respectively (p<0.001). Selected comorbidities in Table 1 are statistically significantly associated with MCI. Current work explores adjusting for age and adding health status indicators: nearly 90% of individuals had BMI data, with mean 28.8 and 30.5 in the MCI and non‐MCI cohorts, respectively (p<0.001); 80% of individuals had smoking status data, 37.6% and 46.9% of the MCI cohort were previous smokers and never smokers, respectively, versus 29.2% and 54.2% in the non‐MCI cohort (all p<0.001).

**Conclusion:**

Consistent with a recent published study, this analysis finds higher unadjusted odds of hypertension, hyperlipidemia, stroke, atherosclerosis, diabetes, weight loss, depression, hearing loss insomnia, sleep apnea, chronic obstructive pulmonary disease, and kidney disease in the MCI cohort. We aim to use the prediction model from this study to improve the ability to discriminate between MCI and non‐MCI individuals and, ultimately, to develop a triage tool for PCPs to identify individuals at elevated MCI risk for further workup, based on routinely available data.